# *Celsr1* suppresses *Wnt5a*-mediated chemoattraction to prevent incorrect rostral migration of facial branchiomotor neurons

**DOI:** 10.1242/dev.200553

**Published:** 2022-11-16

**Authors:** Devynn Hummel, Alexandria Becks, Hongsheng Men, Elizabeth C. Bryda, Derrick M. Glasco, Anand Chandrasekhar

**Affiliations:** ^1^ Division of Biological Sciences and Bond Life Sciences Center, University of Missouri, Columbia, MO 65211, USA; ^2^ Department of Veterinary Pathobiology, University of Missouri, Columbia, MO 65211, USA; ^3^ Department of Biology, Bob Jones University, Greenville, SC 29614, USA

**Keywords:** Facial branchiomotor neuron, Mouse knockout, *Celsr1*, *Wnt5a*, Neuronal migration, Hindbrain explant

## Abstract

In the developing hindbrain, facial branchiomotor (FBM) neurons migrate caudally from rhombomere 4 (r4) to r6 to establish the circuit that drives jaw movements. Although the mechanisms regulating initiation of FBM neuron migration are well defined, those regulating directionality are not. In mutants lacking the Wnt/planar cell polarity (PCP) component Celsr1, many FBM neurons inappropriately migrate rostrally into r3. We hypothesized that *Celsr1* normally blocks inappropriate rostral migration of FBM neurons by suppressing chemoattraction towards Wnt5a in r3 and successfully tested this model. First, FBM neurons in *Celsr1; Wnt5a* double mutant embryos never migrated rostrally, indicating that inappropriate rostral migration in *Celsr1* mutants results from *Wnt5a*-mediated chemoattraction, which is suppressed in wild-type embryos. Second, FBM neurons migrated rostrally toward Wnt5a-coated beads placed in r3 of wild-type hindbrain explants, suggesting that excess Wnt5a chemoattractant can overcome endogenous *Celsr1*-mediated suppression. Third, rostral migration of FBM neurons was greatly enhanced in *Celsr1* mutants overexpressing *Wnt5a* in r3. These results reveal a novel role for a Wnt/PCP component in regulating neuronal migration through suppression of chemoattraction.

## Teaser

**Summary:** Evidence for a previously unreported role for a cell-adhesion receptor in regulating neuronal migration in mice by suppressing attraction for a secreted protein.

## INTRODUCTION

During vertebrate brain development, differentiating neurons frequently move considerable distances in order to establish complex circuits required for cognitive and motor function. Within the hindbrain, facial branchiomotor (FBM) neurons, which innervate the facial and jaw muscles, migrate caudally from rhombomere 4 (r4) to form a motor nucleus in r6 (Chandrasekhar, 2004; Guthrie, 2007; Song, 2007). Several genes, including many encoding core components of the Wnt/planar cell polarity (PCP) pathway (Gray et al., 2011; Butler and Wallingford, 2017), are necessary for caudal migration of FBM neurons (Vivancos et al., 2009; Qu et al., 2010; Glasco et al., 2012; Yang et al., 2014). In contrast, only one gene thus far has been shown to regulate directionality. In mice deficient for the atypical cadherin and PCP component Celsr1, some FBM neurons inappropriately migrate rostrally into r3 and r2 instead of caudally (Qu et al., 2010). Tissue-specific knockouts indicate that *Celsr1* function is not required in FBM neurons (Qu et al., 2010), and that it functions non-cell autonomously within the ventricular zone rostral to r4 to prevent inappropriate rostral migration (Glasco et al., 2016). Dye-labeling experiments suggest that rostral migration of FBM neurons in *Celsr1* mutants is not the consequence of a random loss of cell polarity, but instead results from the loss of a local guidance cue (Glasco et al., 2016). Intriguingly, *Wnt5a*, which encodes a putative chemoattractant of FBM neurons (Vivancos et al., 2009), exhibits an overlapping expression pattern with *Celsr1* in the rostral hindbrain. Here, we test an unconventional model for how *Celsr1* regulates *Wnt5a* to ensure caudal migration of FBM neurons.

## RESULTS AND DISCUSSION

**Fig. 1.**
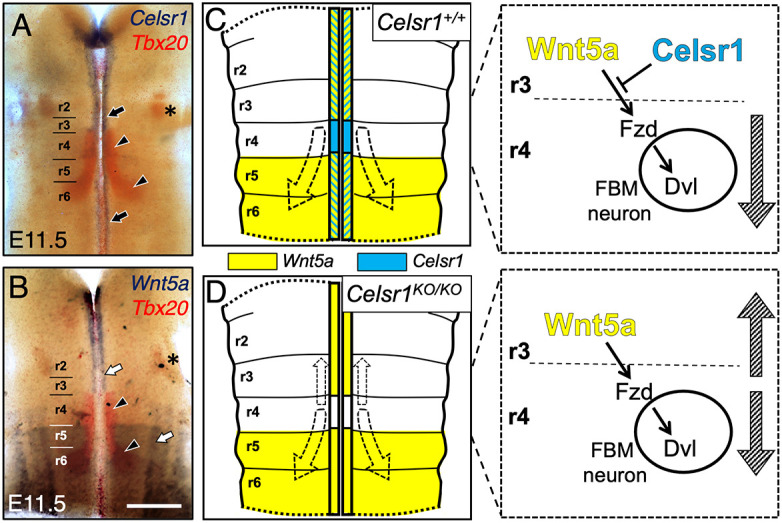
**Model for *Celsr1*-mediated suppression of *Wnt5a* function.** (A,B) Dorsal views of hindbrains processed for two-color *in situ* hybridization. *Tbx20* expression (red) marks FBM neurons in rhombomere 4 (r4) to r6 (arrowheads) and trigeminal motor neurons in r2 (asterisks). (A) *Celsr1* is expressed medially along the entire rostrocaudal extent of the hindbrain (black arrows). (B) *Wnt5a* expression (white arrows) is absent in r4, extends medially up to the r3/r4 boundary and is evident in a broad domain in the caudal hindbrain from the r4/r5 boundary. (C) Schematic of *Celsr1* (blue) and *Wnt5a* (yellow) expression patterns in a wild-type hindbrain and a working model for regulating FBM directionality. The blue/yellow hatched regions indicate overlapping expression of the two genes. Dashed arrows indicate the birth of FBM neurons in r4, caudal migration into r5 and r6, and subsequent dorsolateral migration within r6. In r3, *Celsr1* suppresses *Wnt5a* function and prevents the rostral migration of FBM neurons into r3, resulting exclusively in caudal migration (hatched downward arrow). Fzd, Frizzled receptor; Dvl, Disheveled signaling. (D) In a *Celsr1* knockout (KO), loss of *Celsr1* expression (function) relieves suppression of *Wnt5a* function, leading to rostral migration of some FBM neurons (hatched upward arrow) towards the Wnt5a chemoattractant in the rostral hindbrain. A large number of FBM neurons migrate caudally as in wild type (hatched downward arrow). Scale bar: 400 µm.

**Fig. 2.**
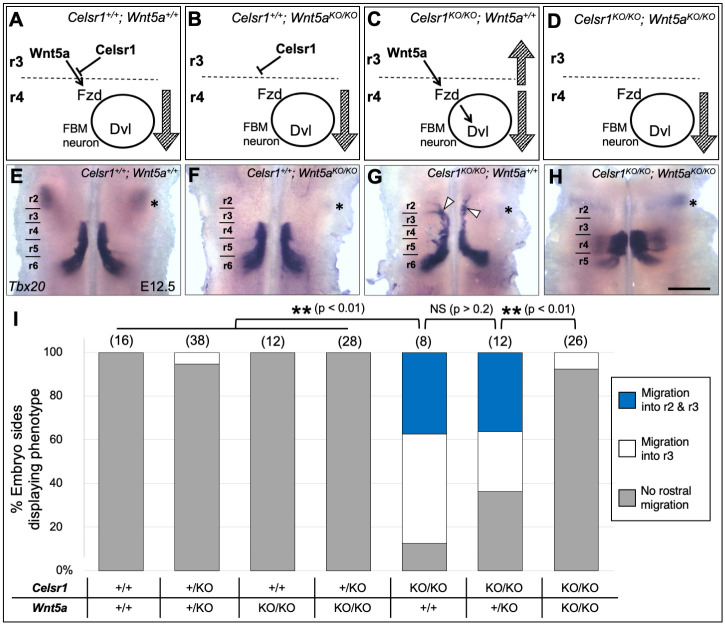
**Rostral migration defect of *Celsr1* mutants is rescued upon knockout of *Wnt5a*.** (A-D) Schematics of our working model of how *Celsr1* and *Wnt5a* function to regulate FBM neuron migration in different genetic conditions. The model predicts that FBM neurons will not exhibit any rostral migration defects in *Celsr1; Wnt5a* double mutants (D). (E-H) Dorsal views of E12.5 hindbrains processed for *Tbx20 in situ* hybridization. Asterisk in each panel indicates the trigeminal motor nucleus in r2. (E) *Tbx20* is expressed in FBM neurons spanning r4 to r6, and the trigeminal motor neurons (asterisk). (F) In *Wnt5a* mutants, all FBM neurons migrate caudally into r5 and r6. (G) In *Celsr1* mutants, a large number of FBM neurons migrate rostrally into r3 and r2 (arrowheads). (H) As predicted, rostral migration is suppressed in double mutants. (I) Quantification of rostral migration phenotypes. The migration phenotypes on the left and right sides of the hindbrain were scored as independent data points because the rostral migration defect in *Celsr1* mutants varied in severity between the two sides. Number of embryo sides in parentheses (double the number of embryos). The differences between genotypes were tested using an unpaired Student's *t*-test. Rostral migration defects were less prevalent in *Celsr1* mutants carrying one copy of *Wnt5a* compared with those with two copies. NS, not significant. Scale bar: 400 µm.

*Celsr1* is expressed in a restricted fashion in the hindbrain at E10.5 and later ages (Fig. S1A) (Qu et al., 2010). At E11.5, *Celsr1* is expressed at all rostro-caudal levels of the hindbrain (Fig. 1A), but only within a small domain of the ventricular zone immediately dorsal and adjacent to the floor plate (Fig. S1C) (Qu et al., 2010). Importantly, *Celsr1* is not expressed in FBM neurons (Fig. 1A), consistent with a non-autonomous role for *Celsr1* in regulating FBM neuron migration (Qu et al., 2010). *Wnt5a* is expressed at a low level and in a rostrally restricted domain at E10.5 (Fig. S1B). This expression pattern is more defined by E11.5, with a rostro-medial domain extending up to the r3-r4 boundary, and a caudal domain evident from the r4-r5 boundary (Fig. 1B; Fig. S1D) (Vivancos et al., 2009; Glasco et al., 2016). These data indicate that *Celsr1* and *Wnt5a* are expressed in overlapping patterns in midline tissues in r3 and r2 before the onset (E10.5) and during the early stages (E11.5) of FBM neuron migration (Fig. 1C; Fig. S1). Even though the rostral *Wnt5a* domain extends up to and abuts the rostral most FBM neurons in r4 (Fig. 1B), these neurons never migrate into r3 (toward the putative chemoattractant) in wild-type embryos but do so extensively in *Celsr1* knockouts (Qu et al., 2010). We propose that putative chemoattraction of FBM neurons located near the r3-r4 boundary toward the rostral source of Wnt5a in r3 is normally suppressed by *Celsr1* (Glasco et al., 2016) (Fig. 1C). In *Celsr1* knockouts, this suppression is relieved, resulting in FBM neurons migrating into r3 and r2 (Fig. 1D; Fig. 2A,C). This model predicts that the rostral migration seen in *Celsr1* single mutants would not occur in *Celsr1; Wnt5a* double mutants because the putative Wnt5a chemoattractive source is lost (Fig. 2D). We have tested and confirmed this prediction (Fig. 2).

E12.5 embryos were collected from *Celsr1^+/KO^; Wnt5a^+/KO^* double heterozygote crosses, genotyped and processed for *Tbx20 in situ* hybridization. The four phenotypic classes were recovered in roughly Mendelian ratios (Fig. S2), with a slight over-representation of double mutants, which often exhibited open neural tubes characteristic of Wnt/PCP mutants such as Looptail (*Vangl2^−/−^*) (Curtin et al., 2003; Glasco et al., 2012). FBM neurons migrated caudally in *Wnt5a^KO/KO^* embryos obtained from single (Fig. S3F,G) or double heterozygote crosses (Fig. 2F,I) in a similar fashion to wild-type embryos (Fig. 2E,I), although there was a slight broadening of the lateral pathway caudal to r5, suggestive of precocious dorsolateral migration (Vivancos et al., 2009). FBM neurons migrated rostrally into r3 and r2 in *Celsr1^KO/KO^; Wnt5a^+/+^* embryos obtained from these crosses (Fig. 2G,I), as in *Celsr1^KO/KO^* embryos obtained from single heterozygote matings (Fig. S3C,G) (Qu et al., 2010). Reducing *Wnt5a* dose by removing one wild-type allele slightly decreased the incidence of rostral migration in *Celsr1^KO/KO^; Wnt5a^+/KO^* embryos (Fig. 2I). Importantly, eliminating *Wnt5a* function almost completely suppressed inappropriate rostral migration in *Celsr1^KO/KO^; Wnt5^KO/KO^* embryos (Fig. 2H,I) as predicted by our model (Fig. 2D). Suppression of rostral migration in double mutants may reflect the inability of FBM neurons to migrate into r3 due to potential defects in the anterior hindbrain of the mutants. However, the efferent neuron marker *Gata3* (Karis et al., 2001) and rhombomere marker *Egr2* (Schneider-Maunoury et al., 1993) were both expressed normally in r3 in double mutants (Fig. S4), ruling out non-specific causes for the loss of rostral migration. We conclude that FBM neurons migrate rostrally in *Celsr1^KO/KO^* embryos due to an unmasking of the Wnt5a chemoattractant in r3.

To test further whether Wnt5a can act as a chemoattractant in the anterior hindbrain, we placed beads coated with recombinant Wnt5a in the rostral hindbrains of E11.5 *SE1::GFP* wild-type explants cultured *in vitro*. Similar to previous observations (Vivancos et al., 2009), FBM neurons migrated rostrally in significant numbers from r4 towards the Wnt5a beads in r3 within 24 h (strong attraction) in the majority of explants (Fig. S5D,G), while PBS-coated beads elicited no effect (Fig. S5B,G). Importantly, the number of explants exhibiting rostral migration (strong attraction) was substantially reduced when cultured from *SE1::GFP Dvl2^KO/KO^* embryos (Fig. S5F,G), bordering on significance (*P*∼0.07), suggesting that the observed rostral migration is dependent on Disheveled (Dvl) function downstream of Wnt5a-Frizzled signaling. The incomplete effect on rostral migration in *Dvl2* mutant explants may reflect functional redundancy with *Dvl1* and *Dvl3*, which are also expressed in E12.5 hindbrains (Wang et al., 2006). We addressed this issue further by comparing rostral migration towards Wnt5a beads in a sensitized background by treating wild-type and *Dvl2* mutant explants with the ROCK inhibitor Y27632, which attenuates Wnt signaling downstream of Dvl and reduces FBM neuron migration towards Wnt5a beads in a similar explant assay (Vivancos et al., 2009). As expected, rostral migration toward Wnt5a beads was strongly reduced in Y27632-treated *Dvl2* mutant explants compared with Y27632-treated wild-type explants and bordered on significance (Fig. S5G).

**Fig. 3.**
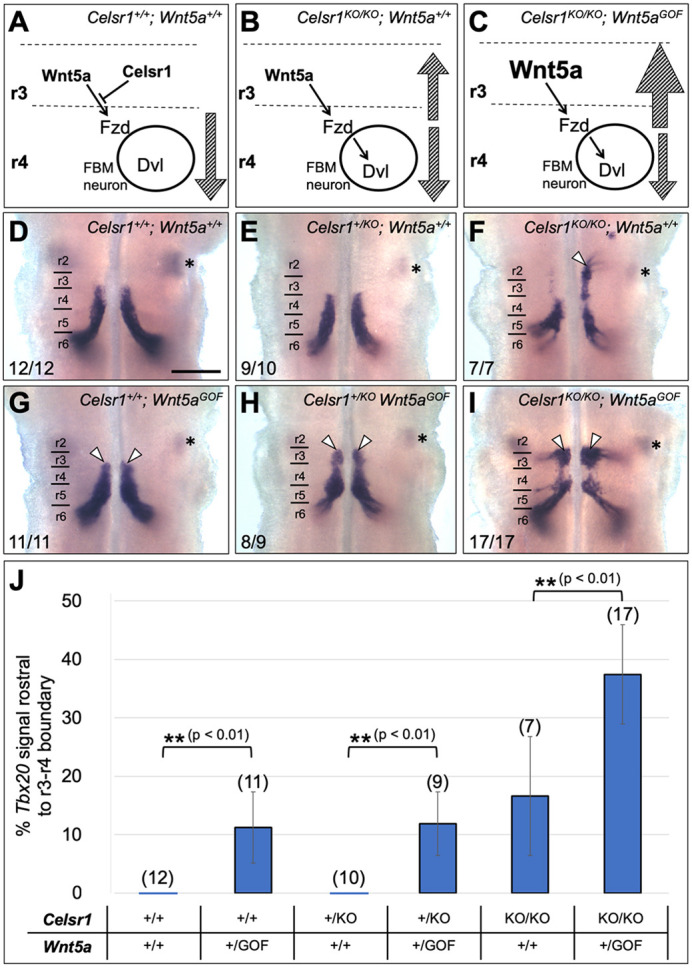
**The *Celsr1* rostral migration phenotype is enhanced upon overexpression of *Wnt5a*.** (A-C) Schematics of our working model of how *Celsr1* and *Wnt5a* function to regulate FBM neuron migration. The model predicts that the rostral migration of FBM neurons into r3 (hatched upward arrows) seen in *Celsr1* mutants (B) will be enhanced by *Wnt5a* overexpression in r3 (C). (D-I) Dorsal views of E12.5 hindbrains processed for *Tbx20 in situ* hybridization. Asterisk in each panel indicates the trigeminal motor nucleus in r2. (D,G) After ectopic expression of *Wnt5a* in r3 in a *Celsr1^+/+^* embryo (G), some FBM neurons (arrowheads) are located in (migrating to) r3. (E,H) *Wnt5a* overexpression in a *Celsr1^+/KO^* embryo (H) results in a large number of FBM neurons located in r3 (arrowheads) compared with a control embryo (E). (F,I) *Wnt5a* overexpression in a *Celsr1^KO/KO^* embryo (I) results in a sharp increase in the number of FBM neurons migrating into r3 (arrowheads) compared with a *Celsr1* mutant embryo (F). (J) Quantification of rostral migration enhancement phenotypes. For each *Ceslr1* genotype, the differences between *Wnt5a* expression conditions were compared using an unpaired Student's *t*-test and found to be significant (**P<0.01) in all cases. Data are mean±s.d. Scale bar: 400 µm.

The rostral migration of FBM neurons in the Wnt5a bead explant experiments is essentially a *Wnt5a* gain-of-function phenotype and is consistent with our model (Fig. 1D), as excess Wnt5a released by the beads presumably overcomes the suppressive effects of *Celsr1* expressed rostral to r4. Therefore, we predicted that *Wnt5a* gain of function in the rostral hindbrain *in vivo* would mimic the bead experiment phenotype, but more importantly enhance the rostral migration of FBM neurons seen in *Celsr1^KO/KO^* embryos (Fig. 3B,C). To generate *Wnt5a* gain of function *in vivo* (*Wnt5a^GOF^*), we employed a *Wnt5a loxP-STOP* allele inserted into the *ROSA26* locus (Cha et al., 2014) crossed to *Egr2-Cre* (Voiculescu et al., 2000), resulting in ectopic and robust *Wnt5a* expression throughout r3 and r5 (Fig. S6C). In *Celsr1^+/+^; Wnt5a^GOF^* embryos, a significant number of *Tbx20*-expressing FBM neurons were located in r3 (Fig. 3G compared with control Fig. 3D). In *Wnt5a^GOF^* embryos, the r2-r3 boundary was demarcated by the caudal edge of the trigeminal motor nucleus, and the rostro-caudal length of r3 was defined as the average length of r3 measured in control embryos (Fig. 3D,E; Fig. S6A,B). In *Celsr1^+/KO^; Wnt5a^GOF^* embryos, rostral migration of FBM neurons was even more evident with neurons spilling over into r2 (Fig. 3H compared with Fig. 3E). In *Celsr1^KO/KO^; Wnt5a^GOF^* embryos, FBM neurons migrate extensively into r3 (Fig. 3I compared with Fig. 3F). Interestingly, although FBM neurons often migrate rostrally into r2 and beyond in *Celsr1^KO/KO^; Wnt5a^+/+^* embryos (Fig. 3F), rostrally migrating neurons, although more numerous, appear to remain largely within r3 in *Celsr1^KO/KO^; Wnt5a^GOF^* embryos (Fig. 3I), likely because r3 is a potent source of Wnt5a chemoattractant due to ectopic expression (Fig. S6C). We quantified the extent of rostral migration in various genotypes (Fig. S7) and noticed a strong additive effect (Fig. 3J). Although ectopic *Wnt5a* expression in r3 did not lead to substantially increased rostral migration in *Celsr1^+/KO^* compared with *Celsr1^+/+^* embryos, there was a dramatic increase in rostral migration in *Celsr1^KO/KO^* compared with *Celsr1^+/KO^* embryos (Fig. 3J), demonstrating that *Celsr1* normally suppresses the chemotactic activity of Wnt5a to prevent inappropriate rostral migration.

Our genetic studies establish definitively a role for Wnt5a as a chemoattractant for FBM neurons. Although previous studies using hindbrain explants and coated beads suggested that Wnt5a may function as a chemoattractant to guide FBM neurons caudally into r5 and r6, the *Wnt5a* knockout phenotype was subtle and suggestive (Vivancos et al., 2009). Our *Wnt5a* loss- and gain-of-function genetic experiments demonstrate conclusively that FBM neurons can be chemoattracted *in vivo* to a Wnt5a source in the rostral hindbrain. However, our data do not provide further insight into whether Wnt5a also functions as a chemoattractant to induce FBM neurons to migrate caudally, as we did not observe pronounced effects on the caudal migratory stream of FBM neurons in the *Wnt5a* loss- and gain-of-function conditions. There was a marked reduction in the size of the caudal migratory stream in *Celsr1^KO/KO^; Wnt5a^GOF^* embryos, likely due to a substantial increase in the number of FBM neurons migrating rostrally into r3. However, caudally migrating neurons did not accumulate preferentially in r5 in *Wnt5a^GOF^* embryos, as might be expected due to overexpression of *Wnt5a* in r5 (Fig. S6C). Importantly, enhancement of Wnt5a-mediated chemoattraction in *Celsr1^KO/KO^; Wnt5a^GOF^* embryos further validated the role of *Celsr1* in suppressing *Wnt5a* function in the rostral hindbrain.

Wnt5a has demonstrated roles in the nervous system as a chemoattractant for hindbrain commissural growth cones acting via Fzd receptors (Lyuksyutova et al., 2003; Onishi et al., 2013) and as a chemorepellent for corticospinal and callosal growth cones via Ryk receptors (Hutchins et al., 2011). We provide strong genetic evidence for a chemoattractive function for Wnt5a in the migration of neuronal cell bodies. Our Wnt5a bead studies suggest that this function depends on Dvl signaling, which is downstream of the Fzd receptor. Although a chemoattractive role for Wnt5a in regulating neuronal migration has not been previously reported, Wnt5a-mediated signaling has been implicated in the migration of several non-neuronal cell types such as primordial germ cells (Laird et al., 2011), cardiomyocytes (Moyes et al., 2013), T-cells (Ghosh et al., 2009), and breast cancer and leukemia cells (Hasan et al., 2017; Kim et al., 2020).

Although Wnt5a is a chemoattractant for FBM neurons, they never migrate rostrally out of r4 towards the source of Wnt5a in r3 in wild-type embryos. Here, we have tested and confirmed that *Celsr1*, the expression of which overlaps that of *Wnt5a* in r3 and r2, suppresses chemoattraction toward Wnt5a, ensuring that FBM neurons exclusively migrate caudally out of r4. The mechanism of suppression of Wnt5a activity or function by Celsr1 remains to be determined, and could be direct or indirect, extracellular or intracellular, and cell-autonomous or non-autonomous. Elucidating this mechanism will be a topic for future study.

## MATERIALS AND METHODS

### Animals

Mouse colony maintenance and embryo collection was carried out using protocols approved by the Animal Care and Use Committee (ACUC) at the University of Missouri, Columbia, MO, USA. In cages used for timed matings, dams were checked every morning; noon on the day that a copulation plug was detected was defined as embryonic day (E) 0.5. Embryos were staged using standard morphological criteria (Nagy, 2003) before fixation.

### Mouse lines and genotyping

The *Celsr1* knockout (KO) line (Ravni et al., 2009), *Tg(Isl1-EGFP)2Slp (SE1::GFP)* transgenic line (Shirasaki et al., 2006) and the *Egr2^tm2(cre)Pch^* (*Egr2-*Cre, previously *Krox20^Cre^*) Cre line (Voiculescu et al., 2000) were cryo-recovered from frozen sperm by *in vitro* fertilization using C57B6NCrl (*Celsr1^KO^* and *SE1::GFP* lines) and C57B6NJ (*Egr2-Cre* line) donor oocytes. Strain B6;01W55-*Wnt5aGOF* carrying the conditional *Wnt5a^GOF^* allele, generated by inserting a floxed *Wnt5a* expression construct at the *ROSA26* locus (Cha et al., 2014), was a kind gift from Dr Terry Yamaguchi (National Cancer Institute-Frederick, MD, USA). B6;129S7-*Wnt5a^tm1Amc^* (*Wnt5a^KO^*) (Yamaguchi et al., 1999) and 129S-*Dvl2^tm1Awb^* (*Dvl2^KO^*) (Hamblet et al., 2002) mice were purchased from Jackson Laboratory. Genotyping of various lines was carried out as previously described (Yamaguchi et al., 1999; Hamblet et al., 2002; Shirasaki et al., 2006; Cha et al., 2014; Glasco et al., 2016).

### *In situ* hybridization

Synthesis of digoxygenin and fluorescein labeled probes and whole-mount *in situ* hybridization was carried out as described previously (Song et al., 2006; Qu et al., 2010; Glasco et al., 2012; Thoby-Brisson et al., 2012). Demarcation of rhombomere boundaries was performed as described previously (Glasco et al., 2012).

### Hindbrain explant culture and bead placement

Explant culture protocol was adapted from a previously described method (Vivancos et al., 2009). Embryos collected at E11.5 were screened for the presence of GFP-expressing FBM neurons, and hindbrains were dissected in cold L-15 media (Gibco, 11415064). As *Dvl2^KO/KO^* embryos display no overt morphological defects (Hamblet et al., 2002), handplates were saved for genotyping. The dissected hindbrains were placed on laminin-coated filters in six-well plates (laminin from Sigma, L2020; 8μm polycarbonate membrane inserts from Corning costar, 3428), neurobasal medium [Gibco, 21103049, supplemented with 0.001% GDNF (R&D Systems, 212-GD), 1% antibiotic antimycotic (Sigma, A5955), 1% GlutaMAX (Gibco, 35050061) and 2% B-27 (Gibco, 17504044)] was added to the well and incubated at 37°C. After a 30-min incubation, Affi-gel blue agarose beads (Bio-Rad, 153-7301), treated overnight with PBS or Wnt5a (R&D Systems, 645-WN), were placed rostral to the r3/r4 boundary by visualizing the FBM neurons in r4. After a 30-min incubation at 37°C, samples were imaged under GFP fluorescence (designated as 0 h). Explants were imaged again at 24 h and 48 h to visualize the distribution of GFP-expressing FBM neurons and compared with the 0 h timepoint. As migration was evaluated before genotyping, rostral migration phenotypes were essentially scored in a blinded fashion. Explants in which FBM neurons migrated rostrally by more than one bead diameter were scored as exhibiting ‘strong attraction’ (to the beads), while explants with FBM neurons less than one bead diameter of the r3/r4 boundary were scored as exhibiting ‘weak attraction’. The ROCK inhibitor experiments were carried out by adding Y27632 (Tocris, 1254 in DMSO, final concentration 15 µM) to the neurobasal explant medium.

### Fiji image processing and quantification

To quantify the proportion of rostral migration observed among embryos with increased *Wnt5a* expression*,* the r3/4 boundary was first estimated based on control samples (non-*Wnt5a* gain of function; wild type and heterozygote for *Celsr1*). All measurements were carried out using the ‘Multi-measure’ function in Fiji (ImageJ) open-source image analysis software. After establishing the r3/4 boundary for each experimental sample, four ROIs were defined by outlining the *Tbx20* signal (specific to FBM neurons) rostral and caudal to the putative r3/4 boundary for the two sides. After exporting measurements of both the area and intensity, the percentage of signal found rostral to the r3/4 boundary was calculated (Fig. S6).

### Statistical analysis

For all comparisons, a one-tailed, unpaired Student's *t*-test with equal variance was used to determine whether differences were statistically significant (*P*<0.01).

## Supplementary Material

10.1242/develop.200553_sup1 Supplementary information
