# Supplementary material for: Celsr1 suppresses Wnt5a-mediated chemoattraction to prevent incorrect rostral migration of facial branchiomotor neurons
Source: Development. 2022 Nov 16;149(22):dev200553. doi: 10.1242/dev.200553 (PMC9845735; doi:10.1242/dev.200553)
Supplement: Supplementary information [file develop-149-200553-s1.pdf]

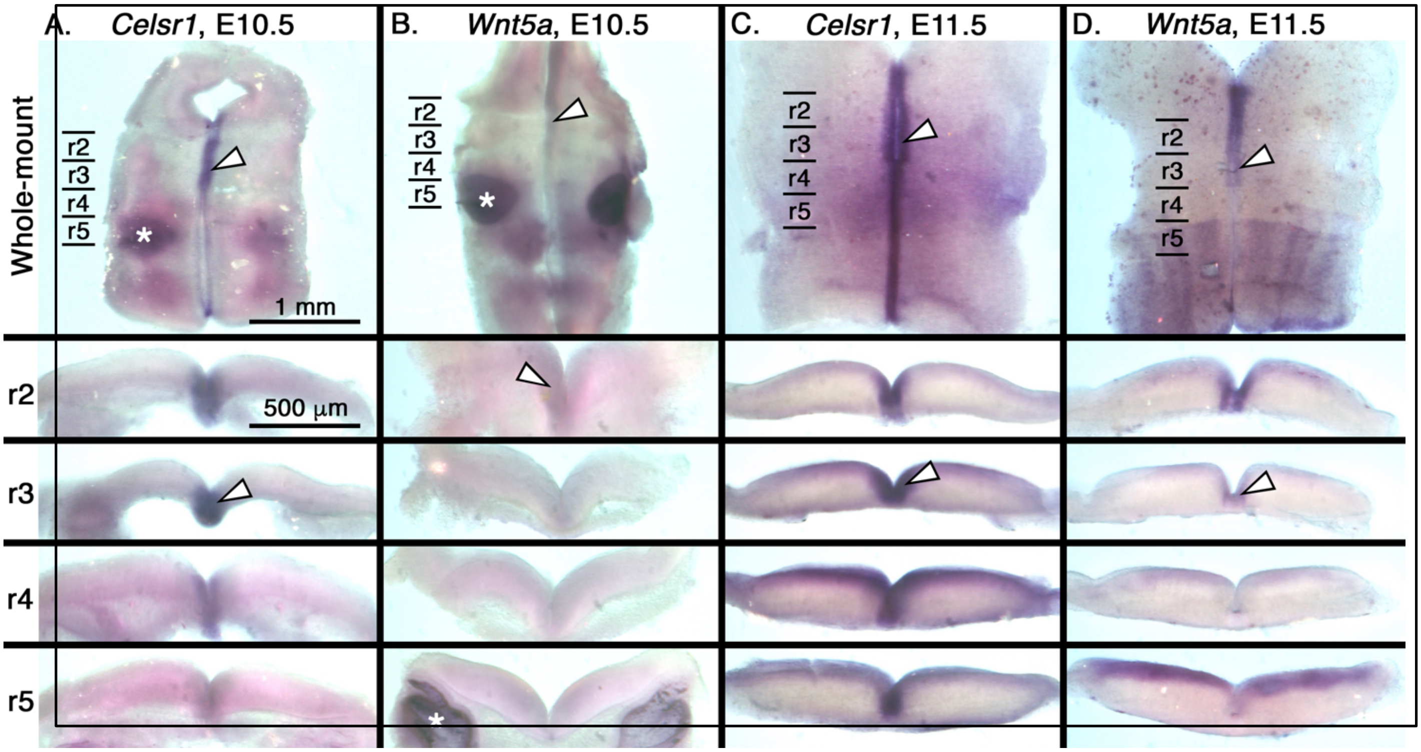

Fig. S1. *Celsr1* and *Wnt5a* are expressed in midline tissues at the onset of migration. Dorsal views of E10.5 (A, B) and E11.5 (C, D) hindbrains processed for *Celsr1* (A, C) and *Wnt5a* (B, D) in situ hybridization. The whole-mounted hindbrains are shown in top panels. Each hindbrain was sectioned by hand to isolate rhombomeres r2 to r5, which were mounted and imaged as cross-sections shown in the lower panels. Asterisks in E10.5 hindbrains indicate non-specific trapping of probe in the otic vesicles. (A, C) *Celsr1* is expressed in midline tissues (arrowheads) at all axial levels (rhombomeres) at both ages, with stronger expression evident in r2 and r3. (B, D) *Wnt5a* is expressed (arrowheads) at a low level in rostral midline tissues of the E10.5 hindbrain, and at a higher level up to the r3/r4 boundary in the E11.5 hindbrain. At E11.5, *Wnt5a* is not expressed in midline tissues in r4 and r5 but is expressed strongly in the ventricular zone in r5 and more posterior rhombomeres. Scale bars: (in A) for A-D whole-mount hindbrain panels, 1000 µm; (in A, r2) for all rhombomere cross-section panels, 500 µm.

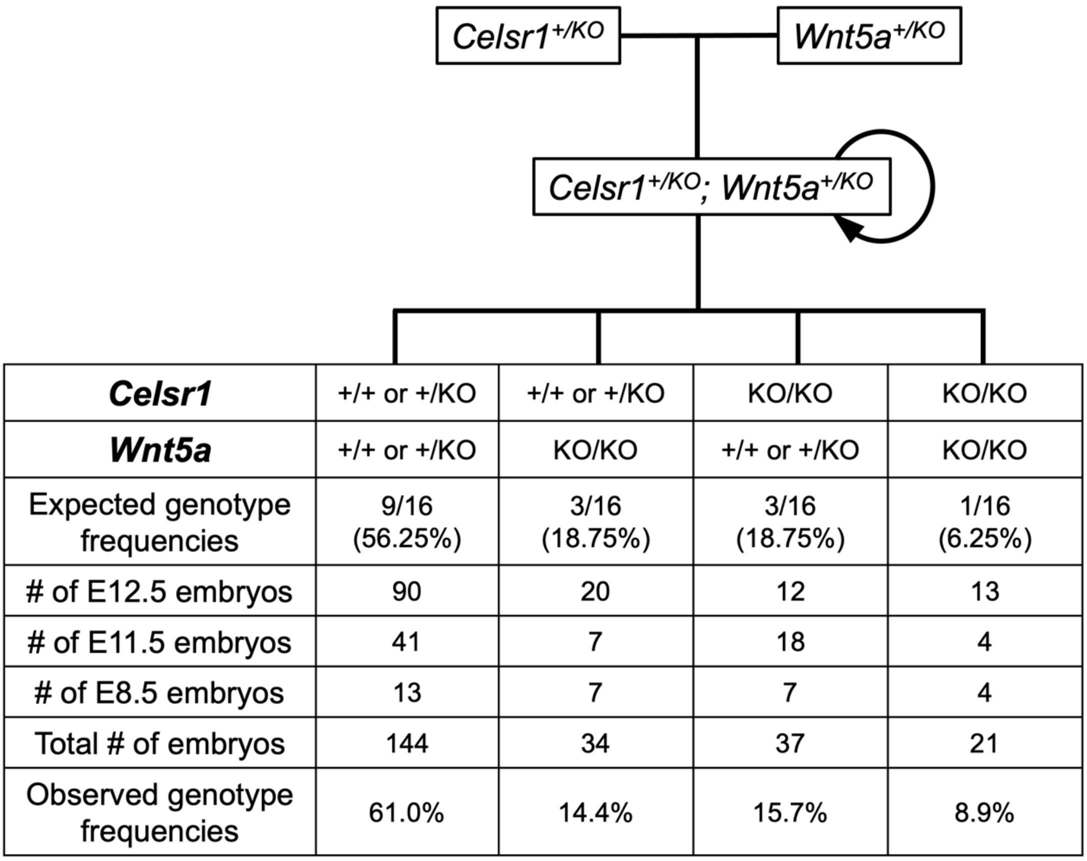

Celsr1	+/+ or +/KO	+/+ or +/KO	KO/KO	KO/KO
Wnt5a	+/+ or +/KO	KO/KO	+/+ or +/KO	KO/KO
Expected genotype frequencies	9/16 (56.25%)	3/16 (18.75%)	3/16 (18.75%)	1/16 (6.25%)
# of E12.5 embryos	90	20	12	13
# of E11.5 embryos	41	7	18	4
# of E8.5 embryos	13	7	7	4
Total # of embryos	144	34	37	21
Observed genotype frequencies	61.0%	14.4%	15.7%	8.9%

Fig. S2. Breeding scheme used to generate *Celsr1; Wnt5a* double mutants.
The observed numbers of embryos obtained for various genotypes (pooled for all ages) approximate Mendelian ratios ruling out developmental lethality or arrest in double mutant embryos.

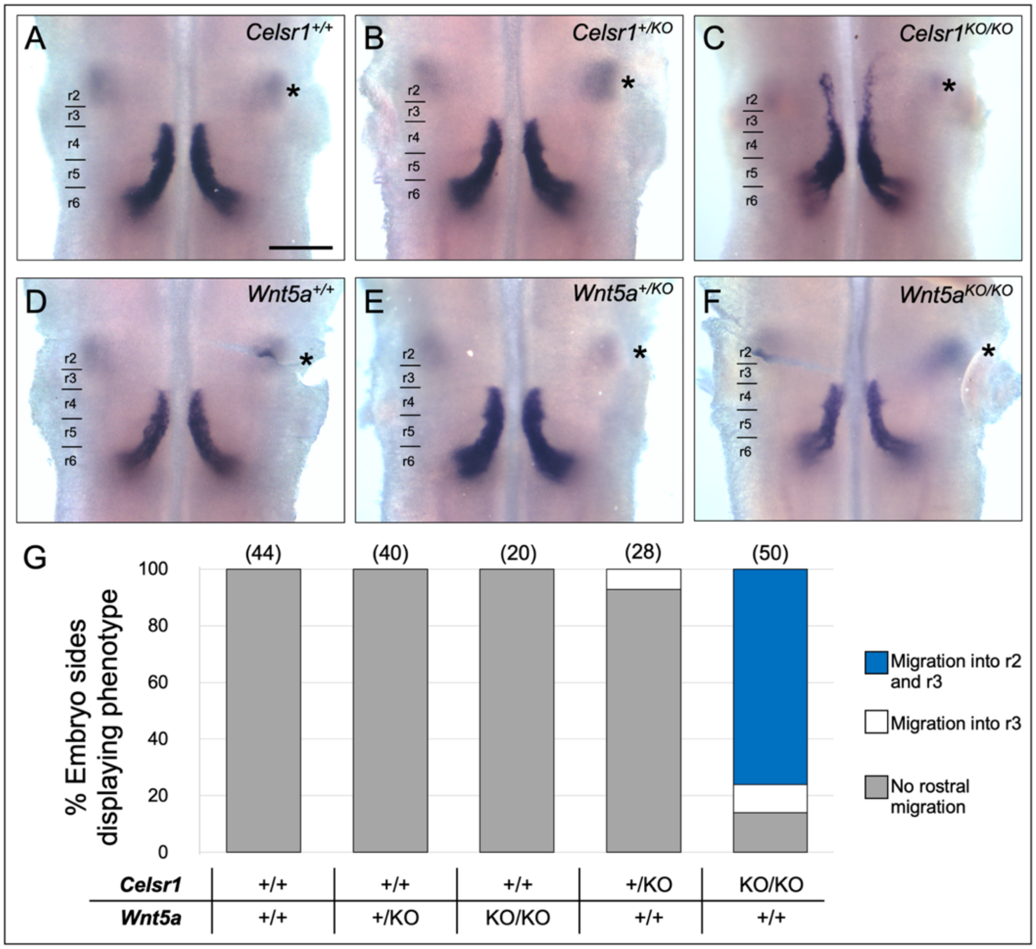

Fig. S3. FBM migration phenotypes in *Celsr1* and *Wnt5a* mutants.

Dorsal views of E12.5 hindbrains processed for *Tbx20* in situ hybridization. *Tbx20* staining marks FBM neurons and trigeminal neurons (asterisk). For all samples, the extent of rostral migration was scored and subsequently quantified (G). While varying numbers of FBM neurons migrated rostrally in nearly all *Celsr1* mutants (C), these neurons never migrated rostrally in *Wnt5a* mutants (F). Rostral migration was scored separately for the left and right sides of the hindbrain due to variable expressivity of the phenotype. Number of embryo sides in parentheses (double the number of embryos). Scale bar (in A) for A-F, 400 μm.

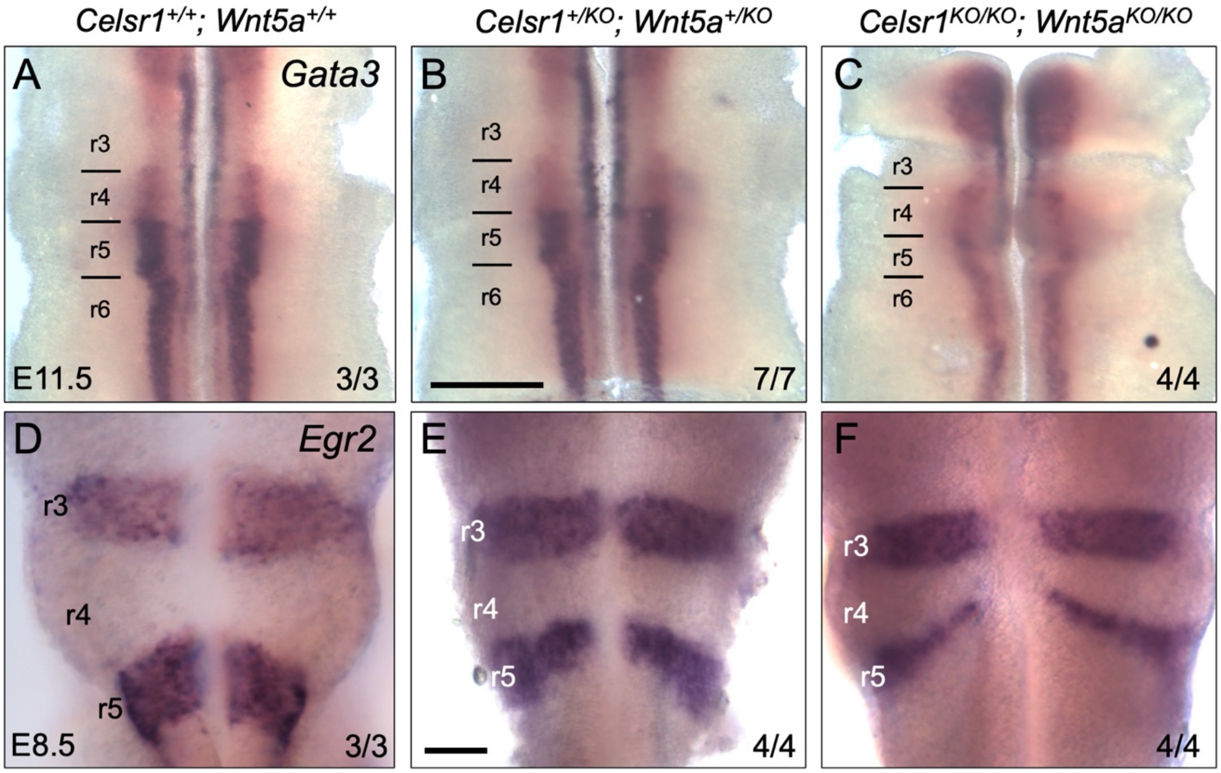

Fig. S4. Rhombomere 3 develops normally in *Celsr1; Wnt5a* double mutant embryos. (A-F) Dorsal views of E11.5 hindbrains processed for *Gata3* in situs (A-C) and E8.5 embryos processed for *Egr2* in situs (D-F). The expression patterns of both genes in various hindbrain rhombomeres, especially r3, were similar between wildtype, double heterozygous and double mutant embryos. Numbers of embryos examined are indicated. Scale bars: (in B) for A-C, 400 μm; (in E) for D-F, 50 μm.

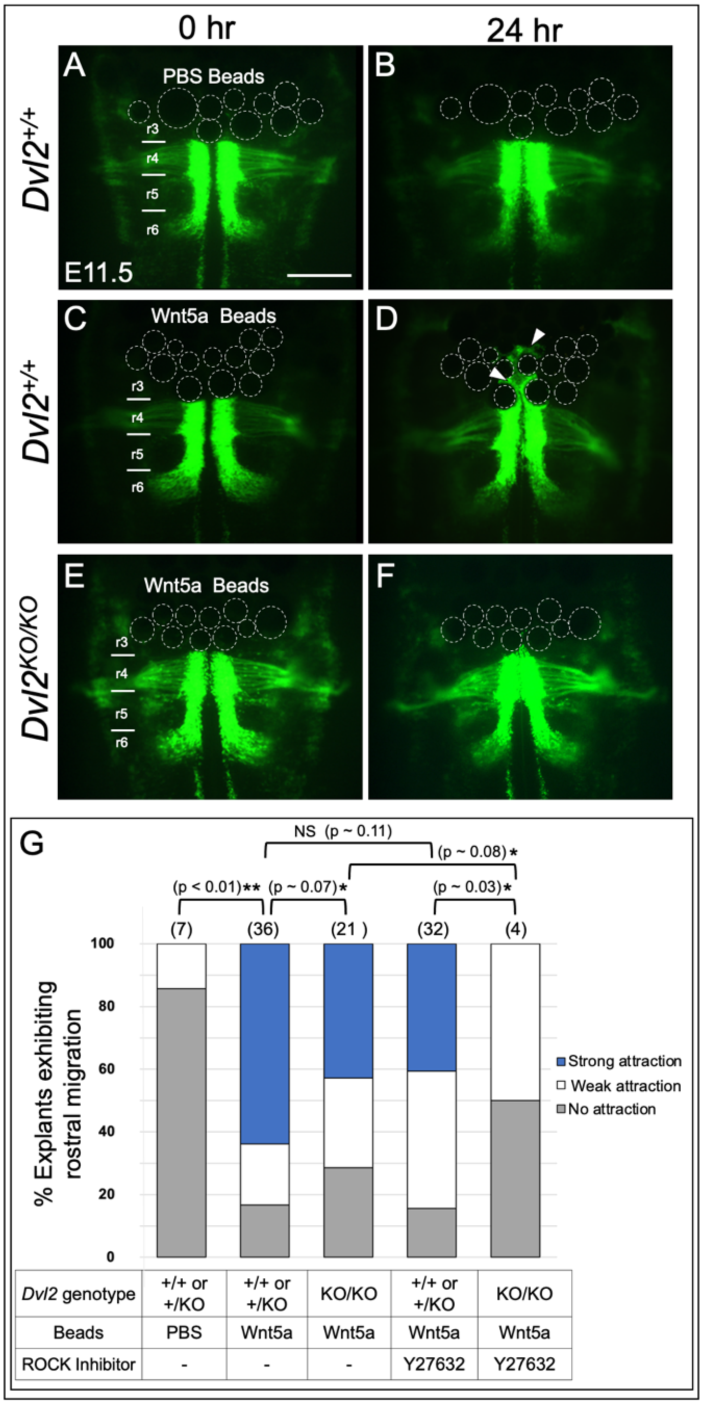

Fig. S5. *Dvl2* mutant FBM neurons exhibit reduced attraction towards Wnt5a-treated beads. Panels A-F show dorsal views of E11.5 hindbrain explants from *SE1::GFP* transgenic embryos of the indicated genotypes immediately (0 hr; A, C, E) or 24 hours (B, D, F) after the

Development • Supplementary information

placement of Wnt5a- or PBS-treated beads. (A, B) PBS-treated beads placed in r3 did not elicit any effect in the GFP-expressing FBM neurons in r4. (C, D) Within 24 hours of placing Wnt5a-treated beads in r3 in a wildtype explant, a large number of FBM neurons (arrowheads) have migrated rostrally 2-3 bead diameters from the r3/r4 boundary (Strong attraction). (E, F) In a *Dvl2* mutant explant, a small number of FBM neurons have breached the r3/r4 boundary by 24 hours, and an even smaller number has migrated rostrally less than a bead diameter (Weak attraction). (G), Quantification of phenotypes. Number of explants in parentheses. Student's t-test indicates that the difference in attraction to Wnt5a beads between wildtype and *Dvl2* mutants is bordering on significance (*). Similarly, treatment of explants with Y27632, a ROCK inhibitor, further attenuates attraction toward Wnt5a beads in *Dvl2* mutant explants, and borders on significance when compared to untreated *Dvl2* mutant explants or inhibitor-treated wildtype explants. Scale bar (in A) for A-F, 600 μm.

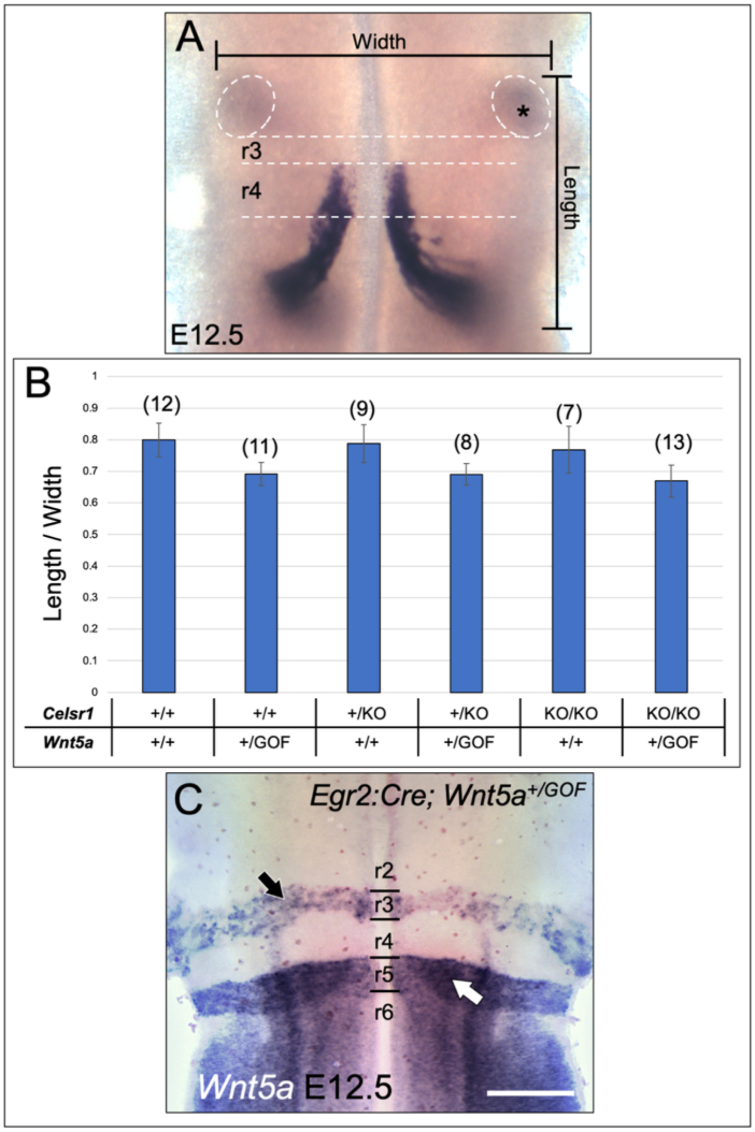

Fig. S6. Hindbrain size and rhombomere dimensions in *Wnt5a*^{GOF} embryos.
(A, C) Dorsal views of E12.5 hindbrains processed for *Tbx20* (A) and *Wnt5a* (C) in situs. (A) Length and width were defined as shown, using as landmarks the outlined edges of the trigeminal motor nuclei in r2 (circled, asterisk) and the caudal edge of the stream of migrating FBM neurons in r6. The boundaries of r3 and r4 were defined for control embryos from the location of the *Tbx20*-expressing trigeminal and FBM neurons and used to establish the corresponding boundaries (and rhombomere lengths) in *Wnt5a*^{GOF} embryos. (B) Hindbrain

Development • Supplementary information

dimensions were relatively constant across all genotypes and *Wnt5a* expression. (C) In an *Egr2-Cre; Wnt5a*$^{+/GOF}$ hindbrain, overexpression of *Wnt5a* is evident in r3 (black arrow) and r5 (white arrow), and r3 is shorter than r4 along the rostrocaudal axis, supporting the measurements in A and B. Scale bar (in C) for A and C, 400 μm.

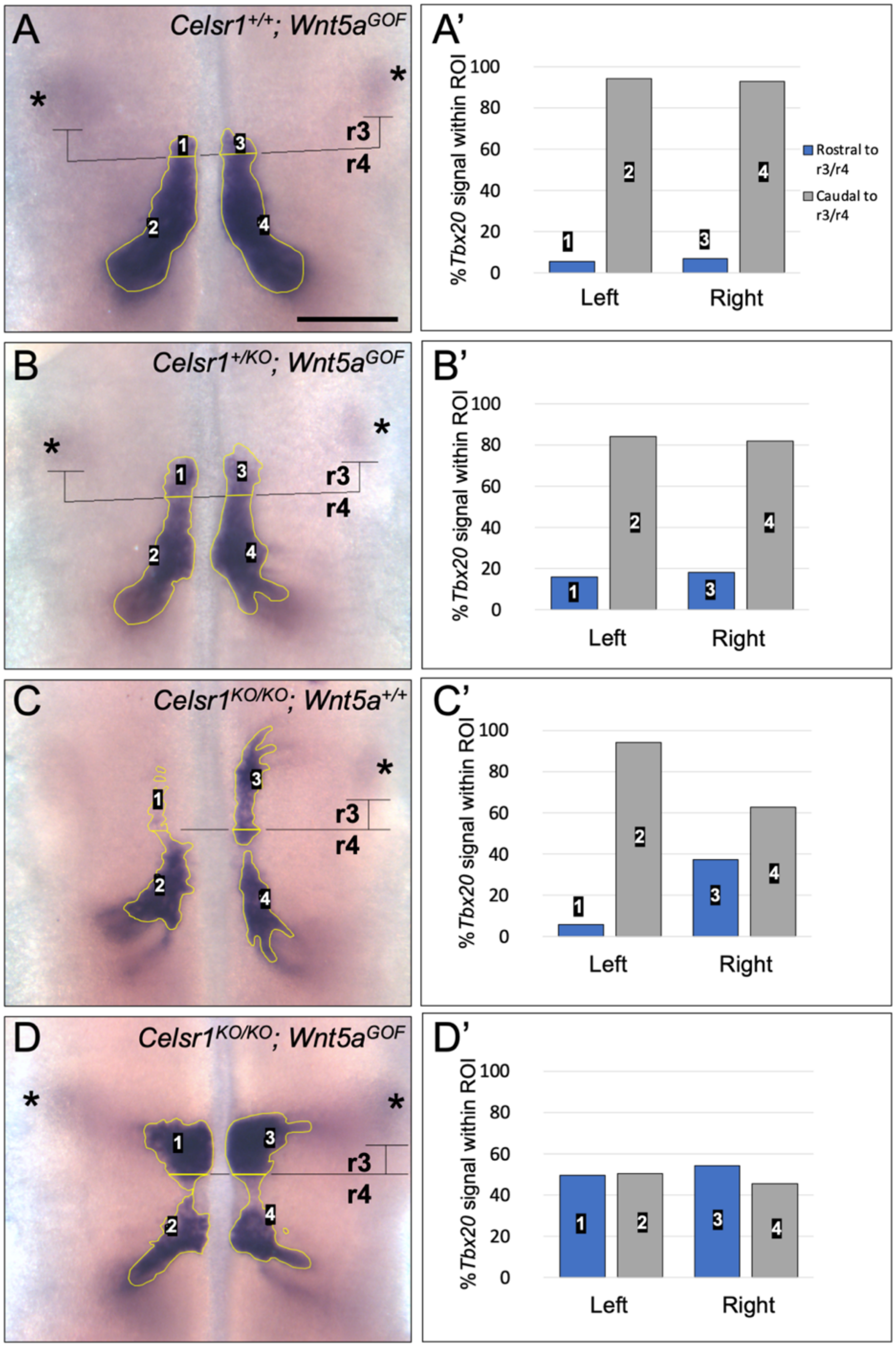

Fig. S7. Quantifying rostral migration of FBM neurons using the Fiji (ImageJ) program. (A-D) Dorsal views of E12.5 hindbrains processed for *Tbx20* in situs. Using the caudal margin of the trigeminal motor nuclei (asterisks), the putative r3/4 boundary was drawn using the

Development • Supplementary information

average length of r3 calculated from control embryos. This enabled demarcation of the *Tbx20*-expressing migratory streams into regions of interest (ROI 1-4). (A'-D') Using Fiji, the areas and intensities in these ROIs were measured and plotted as a function of the total *Tbx20* signal for each side of the hindbrain. These measurements were used to calculate the fraction of total *Tbx20* signal found rostral to the r3-r4 boundary shown in Figure 3. Scale bar (in A) for A-D, 400 μm.
