# Supplementary material for: Celsr1 suppresses Wnt5a-mediated chemoattraction to prevent incorrect rostral migration of facial branchiomotor neurons
Source: Development. 2022 Nov 16;149(22):dev200553. doi: 10.1242/dev.200553 (PMC9845735; doi:10.1242/dev.200553)
Supplement: Supplementary file 1 [file develop-149-200553_review_history.pdf]

Celsr1 suppresses *Wnt5a*-mediated chemoattraction to prevent incorrect rostral migration of facial branchiomotor neurons

Devynn Hummel, Alexandria Becks, Hongsheng Men, Elizabeth C. Bryda, Derrick M. Glasco and Anand Chandrasekhar
DOI: 10.1242/dev.200553

Editor: Steve Wilson

Review timeline

Original submission:	20 January 2022
Editorial decision:	9 March 2022
First revision received:	23 September 2022
Accepted:	20 October 2022

Original submission

First decision letter

MS ID#: DEVELOP/2022/200553

MS TITLE: *Celsr1* Suppresses *Wnt5a*-Mediated Chemoattraction to Prevent Incorrect Rostral Migration of Facial Branchiomotor Neurons

AUTHORS: Devynn Hummel, Alexandria Becks, Hongsheng Men, Elizabeth C Bryda, Derrick M Glasco, and Anand Chandrasekhar

I have now received two referees' reports on the above manuscript, and have reached a decision. The referees' comments are appended below, or you can access them online: please go to BenchPress and click on the 'Manuscripts with Decisions' queue in the Author Area.

As you will see, the referees differ in their opinions on the suitability of your manuscript for publication in Development. They both recognise the quality of the work you present but one reviewer considers that there is notsufficient advance in understanding of migration over what is already published. This reviewer is firmly of the opinion that you need a more thorough understanding of how the molecules you manipulate are involved in the migrations of the FBNs. Both reviewers make suggestions for improving the study. If you are able to revise the manuscript along the lines suggested, I will be happy receive a revised version of the manuscript. Please also note that Development will normally permit only one round of major revision.

We are aware that you may be experiencing disruption to the normal running of your lab that make experimental revisions challenging. If it would be helpful, we encourage you to contact us to discuss your revision in greater detail. Please send us a point-by-point response indicating where you are able to address concerns raised (either experimentally or by changes to the text) and where you will not be able to do so within the normal timeframe of a revision. We will then provide further guidance. Please also note that we are happy to extend revision timeframes as necessary.

Please attend to all of the reviewers' comments and ensure that you clearly highlight all changes made in the revised manuscript. Please avoid using 'Tracked changes' in Word files as these are lost in PDF conversion. I should be grateful if you would also provide a point-by-point response detailing how you have dealt with the points raised by the reviewers in the 'Response to Reviewers' box. If you do not agree with any of their criticisms or suggestions please explain clearly why this is so.

© 2022. Published by The Company of Biologists under the terms of the Creative Commons Attribution License (https://creativecommons.org/licenses/by/4.0/).

Reviewer 1

Advance summary and potential significance to field

The paper shows that Wnt5a expressed in the rostral hindbrain has the capacity to inappropriately attract FBMNs, and demonstrates that such inappropriate migration is countered by Celsr1 activity. While the work is well done, combining compelling double mutant analysis and careful gain of function work, the advance is nevertheless a relatively minor one, as it was already known that inappropriate rostral migration of FBMNs occurs both in Celsr1 mutants and in response to ectopic Wnt5a.

Comments for the author

This short paper evaluates the hypothesis that inappropriate rostral migration of FBMNs in the hindbrain of Celsr1-deficient mouse is Wnt5a dependent. The authors begin their study by demonstrating that while Celsr1 mutants show rostral FBMN migration (in accord with previous findings) this aberrant migration no longer occurs when Wnt5a is also mutated. The double mutant analysis is compelling.

The underlying model is that Wnt5a functions as a chemoattractant for FBMNs. That Wnt5a is capable of playing such a role was already demonstrated by Vivancos et al. in 2009, and this manuscript repeats the key 2009 demonstration that rostrally placed Wnt5a beads attract FBMNs. The authors demonstrate that Wnt5a is expressed in a manner that allows this role, and make efforts to build on the previous work in a couple of different ways.
First, they use a Dvl2 mutant background to ask whether the rostral FBMN migration is Disheveled2 dependent. The data (Fig. 3E-G) are weak: although 21 samples is a reasonable 'n', there is only a subtle trend rather than any significant difference when Dvl2 is knocked out. The authors suggest this is due to redundancy with other Dvl molecules, which is likely correct, but overall, this experiment is unsatisfying as it makes a negligible addition to our understanding.
The second new approach is to build on the previous bead experiments by developing a gain-of-function (GOF) approach in which Wnt5a is expressed at high levels in r3/r5 using an Egr2Cre driver. The r3 expression of Wnt5a alone causes some minor rostral FBMN migration and in a Celsr1 mutant background induces strong rostral migration. These findings are consistent with the bead experiments and further bolster the conclusion that Wnt5a can act as a rostral chemoattractant for FBMNs.
Interestingly, despite elevated expression of Wnt5a in r5, there is no change in behavior of the caudal migrating FBMNs. If Wnt5a were a key chemoattractant needed for 'normal' caudal migration, then FBMNs might arguably be expected to pile up in r5 and not continue on. This lack of a GOF phenotype in caudal migration is consistent with the very minor deficits found in caudal FBMN migration in Wnt5a mutants (Fig. 2).
In summary, the data nicely support a model in which rostral Wnt5a has the capacity to inappropriately attract FBMNs and this inappropriate migration is countered by Celsr1 activity. The data do not suggest Wnt5a is also needed for normal migration, and to their credit the authors have carefully not over sold their findings, with the title of the paper accurately capturing their main finding.
The strengths of this manuscript are that the double mutant analysis has confirmed the interaction between Wnt5a and Celsr1, and the gain of function work has added additional support for a chemoattractive capability of Wnt5a. However, given the unsatisfying Dvl2 experiments no deeper molecular understanding of how rostral migration is caused or controlled has been achieved, and there is also no addition to our understanding of "normal" caudal migration. Thus, while the work is carefully done and the figures are of high quality, as it currently stands this paper offers an insufficient advance to warrant publication at Development. If the authors were able to extend their study to add insights into how Wnt signaling causes cells to migrate in a particular direction, the paper would become more appropriate for this journal.

Minor Points.
1. Abstract: While the sentence on lines 34/35 ('FBM neurons in Celsr1; Wnt5a double mutant embryos never migrated rostrally, indicating that suppression of Wnt5a-mediated chemoattraction was relieved in Celsr1 mutants.') is correct, it is challenging to parse out before reading more of the paper and could perhaps be rephrased for clarity.

© 2022. Published by The Company of Biologists under the terms of the Creative Commons Attribution License (https://creativecommons.org/licenses/by/4.0/).

2. The Fig. 1 legend needs to clarify that green hatching represents the dual expression domain.

Reviewer 2

Advance summary and potential significance to field

In this work, the authors show convincingly that the PCP component Celsr1 prevents incorrect rostral migration of FBM neurons through suppression of Wnt5a-mediated chemoattraction. FBM neurons are generated in r4 and undergo a caudal migration from r4 to r6, which is a unique and intriguing example of neuronal migration. This study is important since that Celsr1is the only one gene that has been shown to regulate directionality of FBM neuron migration, but the mechanism is still unknown, whereas this paper shows clearly a novel model to explain the mechanism. The authors first show that no rostral migration of FBM neurons was observed in Celsr1;Wnt5a double mutant, then illustrate that Wnt5a functions as a chemoattractant to FBM neurons by hindbrain explant and Wnt5a GOF experiments. I find that the experimental design makes sense and the results are convincing. The manuscript is well-written, the conclusions are appropriate and make an important progress to the field.

Comments for the author

Minor Comments:
1. The FBM neurons already start their rostral migration in Celsr1 mutants at E11.5, what's the expression pattern of Celsr1 and Wnt5a in the hindbrain at E10.5 before the neurons start to migrate? It would also be nice to show the Celsr1 and Wnt5a expression not only in flat-mounted samples, but also in hindbrain coronal section at different rhombomere levels of E10.5 and E11.5 embryos.
2. In Celsr1;Wnt5a double mutant, there is no rostral migration of FBM neurons but the caudle migration of FBM neurons is also reduced and can only reach r5 instead of r6 (compare Fig.2H to 2G). What are the possible expiations for this?
3. In Celsr1 knockout hindbrains, abnormal lateral migration in r4 can also be observed sometimes (Qu et al., 2010), this should not be related to Wnt5a since Wnt5a is not expressed in r4. It looks like there is still some lateral migration in r4 of Celsr1;Wnt5a double mutant (Fig. 2H), maybe the authors could quantify or discuss about it.

First revision

Author response to reviewers' comments

We thank the reviewers for their thoughtful comments. We respond below to specific issues (reviewer comment in ***bold***).

Reviewer 1:
1) "While the work is well done, combining compelling double mutant analysis and careful gain of function work, the advance is nevertheless a relatively minor one, as it was already known that inappropriate rostral migration of FBMNs occurs both in Celsr1 mutants and in response to ectopic Wnt5a."
We showed previously that FBM neurons inappropriately migrated rostrally in *Celsr1* mutants (Qu et al., 2010). Building on this study, it took huge amount of additional experiments and six years for us to even propose a model for the underlying cause by postulating a role for Wnt5a (Glasco et al., 2016). It has subsequently involved a large amount of "compelling double mutant analysis and careful gain of function work" (as recognized by the reviewer) described in this manuscript to demonstrate a role for *Celsr1* in suppressing chemoattraction of FBM neurons towards rhombomere 3. A model, however feasible it may be, remains speculative until experimentally tested and validated. Therefore, demonstrating a role for *Celsr1* in suppressing Wnt5a-mediated chemoattraction is a major advance that can serve as a framework for understanding *Celsr1*'s

© 2022. Published by The Company of Biologists under the terms of the Creative Commons Attribution License (https://creativecommons.org/licenses/by/4.0/).

functions in other tissues. Furthermore, since some researchers in the field favor a Wnt/planar cell polarity-based mechanism to underlie FBM neuron migration, providing definitive in vivo evidence for a chemoattraction-based mechanism is a major advance.

We agree with the reviewer that Vivancos et al. (2009) demonstrated that Wnt5a- soaked beads placed laterally within r4 of hindbrain explants can attract FBM neurons to this ectopic location. We also acknowledge that the ex vivo bead-explant assay evidence provided in that paper was instrumental for proposing our mechanistic model of *Celsr1*- mediated suppression. However, *Wnt5a* knockout analyses in the same paper suggested only a minor role in vivo for Wnt5a acting in r5 and r6 to chemoattract and induce FBM neurons to migrate caudally. By contrast, our *Celsr1; Wnt5a* double knockout and *Wnt5a* GOF experiments provide compelling in vivo evidence for a definitive role for Wnt5a in functioning as a chemoattractant at the r3/r4 boundary such that FBM neurons can overcome the suppressive effects of *Celsr1* to migrate toward a Wnt5a source in r3, representing a significant advance in our understanding of the migration process.

2) "The underlying model is that Wnt5a functions as a chemoattractant for FBMNs."

Respectfully, we disagree. The underlying model tested here is that *Celsr1* suppresses Wnt5a from functioning as a chemoattractant for FBM neurons in the rostral hindbrain. The double knockout (Fig. 2) and GOF (current Fig. 3) studies provide strong evidence of this role for *Celsr1*.

3) The strengths of this manuscript are that the double mutant analysis has confirmed the interaction between Wnt5a and Celsr1, …"

We proposed for the first time (Glasco et al., 2016) that *Celsr1* may suppress the function of *Wnt5a* in regulating FBM neuron migration to explain the conditional knockout data reported there. We are not aware of any other studies that have postulated a functional interaction between *Wnt5a* and *Celsr1* in the hindbrain or other tissues. Therefore, it is unclear what the reviewer is referring to when they state that the double mutant analysis (Fig. 2) has confirmed the interaction between *Wnt5a* and *Celsr1*; we first proposed this putative interaction (Glasco et al., 2016), and now we present compelling genetic evidence for it. This is a novel finding and not merely confirming something that was already known from other studies.

4) "The strengths of this manuscript are that .... the gain of function work has added additional support for a chemoattractive capability of Wnt5a."
The main objective of the GOF experiments was not to provide additional support for the chemoattractive capability of Wnt5a. Rather, the experiments *employed the chemoattractive ability of Wnt5a* to test whether excess Wnt5a could overcome the suppressive effects of *Celsr1* to induce FBM neurons to migrate rostrally into r3. The results show conclusively that removing functional copies of *Celsr1* in a *Wnt5a* GOF condition sharply increases the extent of rostral migration.

5) "However, given the unsatisfying Dvl2 experiments no deeper molecular understanding of how rostral migration is caused or controlled has been achieved, ……"

The reviewer has noted two separate issues (indicated by separate underlines above).
i) We agree that the *Dvl2* experiment (original Fig. 3) is unsatisfying but it is not critical to the central finding, namely the ability of *Celsr1* to block *Wnt5a* function (Figs. 2 and 4 of original manuscript). The incomplete effect in *Dvl2* mutant explants is likely due to redundancy with *Dvl1*. To address this further, we used a Wnt pathway ROCK inhibitor identified by Vivancos et al. (2009) in an attempt to sensitize the background and generate a stronger phenotype in *Dvl2* mutants. (i.e., we examined migration toward Wnt5a beads in *Dvl2* mutant explants treated with ROCK inhibitor Y27632). While the inhibitor data and statistics are consistent with our model, the conclusions cannot be definitive due to the small sample size of Y27632-treated mutant explants. Therefore, we have removed original Figure 3 from the main body of the manuscript as it is not crucial for our central conclusions. We have placed it in supplementary data (Figure S5) for completeness and because it represents a substantial amount of work performed by the PhD student, Devynn Hummel.

© 2022. Published by The Company of Biologists under the terms of the Creative Commons Attribution License (https://creativecommons.org/licenses/by/4.0/).

ii) We respectfully disagree. The major finding is that *Celsr1* suppresses *Wnt5a* function to normally (i.e., in wildtype) block rostral migration of FBM neurons. This result is the successful experimental validation of a hypothetical model of the functional interaction between *Wnt5a* and *Celsr1*, and indeed represents a deeper understanding of the mechanisms. We agree that additional molecular insights into the putative interaction between *Celsr1* and *Wnt5a* can be obtained (see #7 below), but our findings provide novel insights into why FBM neurons normally do not migrate rostrally.

6) "However, given the unsatisfying Dvl2 experiments.......and there is also no addition to our understanding of "normal" caudal migration."

Our studies were designed to test a mechanism proposed to explain why FBM neurons do NOT migrate in the "abnormal" rostral direction. By successfully validating this mechanism (*Celsr1* suppresses *Wnt5a*-mediated chemoattraction), we have indeed generated a more complete understanding of the migratory process in the "normal" (wildtype) condition. The conventional view is that neurons or growth cones, or cells in general, migrate in a specific direction toward a chemoattractant or away from a chemorepellent. We must now also consider that neurons can be prevented from migrating in an abnormal direction by suppressing the function of a chemoattractant. The reviewer is correct that our study to understand the mechanisms underlying the abnormal rostral migration in *Celsr1* mutants does not add details to any mechanism of normal caudal migration. However, this is to be expected because caudal migration occurs normally in *Celsr1* mutants; instead they exhibit abnormal rostral migration into which we have now provided novel insight.

7) If the authors were able to extend their study to add insights into how Wnt signaling causes cells to migrate in a particular direction, the paper would become more appropriate for this journal.

We are just as interested as the reviewer in following up on the results of the current study to understand how *Celsr1* suppresses Wnt signaling at the molecular and cellular levels to block inappropriate rostral migration in wildtype embryos. However, the reviewer's suggestion to perform additional studies, without identifying any specific experiment, to make the paper more suitable for the journal is not reasonable for two reasons.

i) As stated in the "Aims and scope" page of the journal, "*Research papers (articles and reports) should therefore pose and test a significant hypothesis or address a significant question, and should provide novel perspectives that advance our understanding of development.*" As explained in responses to other comments from this reviewer, our paper poses and tests a significant hypothesis (Does *Celsr1* suppress *Wnt5a*-mediated chemoattraction?) and provides a novel perspective on understanding neuronal migration (Neurons may migrate in a "correct" direction by being prevented from migrating in the "wrong" direction). Based on these criteria, we believe that our report in its current form is appropriate for the journal.

ii) Without identifying a specific experiment, it is hard to know what the reviewer might consider sufficient to meet their bar for this paper to become suitable. Moreover, experiments testing whether Celsr1 and Wnt5a act in the same or different cells, act cell autonomously or non-autonomously, act extracellularly or intracellularly, and whether they bind directly or indirectly to each other, etc. represent a completely new project requiring several years of funding and experimentation and are simply beyond the scope of the current work.

Minor Points

8) "Abstract: While the sentence on lines 34/35 ('FBM neurons in Celsr1; Wnt5a double mutant embryos never migrated rostrally, indicating that suppression of Wnt5a-mediated chemoattraction was relieved in Celsr1 mutants.') is correct, it is challenging to parse out before reading more of the paper and could perhaps be rephrased for clarity."

© 2022. Published by The Company of Biologists under the terms of the Creative Commons Attribution License (https://creativecommons.org/licenses/by/4.0/).

Done. The rephrased sentence reads "*First, FBM neurons in Celsr1; Wnt5a double mutant embryos never migrated rostrally, indicating that inappropriate rostral migration in Celsr1 mutants results from Wnt5a-mediated chemoattraction, which is suppressed in wildtype embryos*."

9) "The Fig. 1 legend needs to clarify that green hatching represents the dual expression domain."

Done. The text now reads "*Schematic of Celsr1 (blue) and Wnt5a (yellow) expression patterns in a wildtype hindbrain and working model for regulating FBM directionality. The blue/yellow hatched regions denote overlapping expression of the two genes*."

Reviewer 2:

1) "This study is important since that Celsr1is the only one gene that has been shown to regulate directionality of FBM neuron migration, but the mechanism is still unknown, whereas this paper shows clearly a novel model to explain the mechanism."

We agree.

2) "I find that the experimental design makes sense and the results are convincing. The manuscript is well-written, the conclusions are appropriate and make an important progress to the field."

We agree.

Minor Comments

3) "The FBM neurons already start their rostral migration in Celsr1 mutants at E11.5, what's the expression pattern of Celsr1 and Wnt5a in the hindbrain at E10.5 before the neurons start to migrate? It would also be nice to show the Celsr1 and Wnt5a expression not only in flat-mounted samples, but also in hindbrain coronal section at different rhombomere levels of E10.5 and E11.5 embryos."

Done. We performed both of these analyses and have included the in situ data as Supplementary data in new Supplementary Figure 1.

In Fig. 1 of Qu et al. (2010), Celsr1 protein expression was described in E10.5 and E11.5 embryos in r4, and *Celsr1* RNA expression was described in some rhombomeres at E10.5 and E12.5. By performing the additional in situs and cross-sections suggested by the reviewer, we have been able to verify that *Celsr1* and *Wnt5a* are expressed in overlapping patterns in the rostral hindbrain before the onset (E10.5) and the early stages (E11.5) of FBM neuron migration. New text has been added in the Results and Discussion section describing Figure S1, as well as a new Figure legend.

4) "In Celsr1;Wnt5a double mutant, there is no rostral migration of FBM neurons, but the caudle migration of FBM neurons is also reduced and can only reach r5 instead of r6 (compare Fig.2H to 2G). What are the possible expiations for this?"

Potential explanations for this phenotype include: a) non-specific effect due to failure of the neural tube to fully close, a phenotype seen in most of the double mutant embryos, and b) non-specific effect due to a shortening (thinning) of r5 (see Fig. S4). However these reasons are speculative and related to caudal migration, which is not the focus of this report. Therefore, we have not included them in our discussion in the revised manuscript.

5) "In Celsr1 knockout hindbrains, abnormal lateral migration in r4 can also be observed sometimes (Qu et al., 2010), this should not be related to Wnt5a since Wnt5a is not expressed in r4. It looks like there is still some lateral migration in r4 of Celsr1;Wnt5a double mutant (Fig. 2H), maybe the authors could quantify or discuss about it?"

© 2022. Published by The Company of Biologists under the terms of the Creative Commons Attribution License (https://creativecommons.org/licenses/by/4.0/).

Lateral migration of FBM neurons in r4 may reflect precocious differentiation (and resulting dorsolateral migration) of these neurons in *Celsr1 KO/KO* embryos. However, abnormal lateral migration was only rarely observed in r4, and occasionally in r5, in *Celsr1* knockout hindbrains in the original study (see Fig. 2B and F from Qu et al., 2010). Importantly, this phenotype was extremely rare, if at all present, in *Celsr1 KO/KO* embryos (n>50) obtained from the mice maintained in our facility for over 10 years. This difference in migration phenotypes between *Celsr1* knockouts obtained in the Tissir (Qu et al., 2010) and Chandrasekhar lab (this report) may be the result of genetic background differences introduced when we crossed the mutant line into the *SE1::GFP* background in our lab.

Therefore, we are unable to quantify this phenotype.

The lateral migration seen in r4 of *Celsr1; Wnt5a* double mutants is also seen in other mice such as *Vangl2 Looptail* mutants and compound heterozygotes of *Vangl2* and genes like *Celsr1* and *Dvl2* (see Fig. 2I and L, and Fig. 7F, G, and K of Glasco et al., 2012). A feature common to all these genetic combinations is an open neural tube, suggesting that the lateral migration in r4 may be a non-specific effect. Since this report is focused on addressing the mechanisms regulating rostral migration, we have not included a discussion of lateral migration in r4 in the revised manuscript.

Second decision letter

MS ID#: DEVELOP/2022/200553

MS TITLE: *Celsr1* Suppresses *Wnt5a*-Mediated Chemoattraction to Prevent Incorrect Rostral Migration of Facial Branchiomotor Neurons

AUTHORS: Devynn Hummel, Alexandria Becks, Hongsheng Men, Elizabeth C Bryda, Derrick M Glasco, and Anand Chandrasekhar
ARTICLE TYPE: Research Report

I am happy to tell you that the reviewers are happy with your revisions and your manuscript has been accepted for publication in Development, pending our standard ethics checks. The referee reports on this version are appended below.

Reviewer 1

Advance summary and potential significance to field

The authors pose and test the hypothesis that Celsr1 suppresses Wnt5a-mediated chemoattraction of FBMNs in an inappropriate direction. The work is important because it reveals that an important control point in ensuring proper neuronal migration can be the prevention of migration in the wrong direction. Viewing the manuscript though this lens, I conclude that there is important new information here that adds to our understanding of neuronal migration in broad terms.

Comments for the author

This paper was already well written, but now the authors have clarified several issues in the text and made a change in structure that leads to a pleasingly clean and well articulated argument. In responding to reviewer comments, they have also added a thoughtful new experiment in which they interrogate a role for Dvl2 in a background sensitized by use of a ROCK inhibitor. While this additional data does strengthen the model that Wnt5a signaling is Dvl2 mediated, I concur that shifting this still 'non definitive' data into the supplemental information is a good choice to ensure a streamlined story. No further revisions are requested.

© 2022. Published by The Company of Biologists under the terms of the Creative Commons Attribution License (https://creativecommons.org/licenses/by/4.0/).

Reviewer 2

Advance summary and potential significance to field

This paper shows clearly a novel model that Celsr1 suppresses Wnt5a from functioning as a chemoattractant for FBM neurons in the rostral hindbrain.

Comments for the author

The authors have addressed all my comments and the paper is suitable for publication now.

© 2022. Published by The Company of Biologists under the terms of the Creative Commons Attribution License (https://creativecommons.org/licenses/by/4.0/).
